# Cancer and diabetes: the interlinking metabolic pathways and repurposing actions of antidiabetic drugs

**DOI:** 10.1186/s12935-021-02202-5

**Published:** 2021-09-17

**Authors:** Ahmed Olatunde, Manisha Nigam, Rahul Kunwar Singh, Abhaya Shikhar Panwar, Abdulwahab Lasisi, Fahad A. Alhumaydhi, Vijay Jyoti kumar, Abhay Prakash Mishra, Javad Sharifi-Rad

**Affiliations:** 1 Department of Biochemistry, Abubakar Tafawa Balewa University, Bauchi, 740272 Nigeria; 2 Department of Biochemistry, School of Life Sciences, Hemvati Nandan Bahuguna Garhwal University, Srinagar, Garhwal, Uttarakhand 246174 India; 3 Department of Microbiology, School of Life Sciences, Hemvati Nandan Bahuguna Garhwal University, Srinagar, Garhwal, Uttarakhand 246174 India; 4 Maidstone and Tunbridge Wells NHS Trust, Hermitage Lane, Maidstone, Kent, ME169QQ UK; 5 Department of Medical Laboratories, College of Applied Medical Sciences, Qassim University, Buraydah, Saudi Arabia; 6 Department of Pharmaceutical Sciences, Hemvati Nandan Bahuguna Garhwal University, Garhwal, Srinagar, Uttarakhand 246174 India; 7 Department of Pharmacology, School of Clinical Medicine, Faculty of Health Science, University of Free State, 205, Nelson Mandela Drive, Park West, Bloemfontein, 9300 South Africa; 8 Phytochemistry Research Center, Shahid Beheshti University of Medical Sciences, Tehran, Iran

**Keywords:** Cancer, Diabetes, Repurposing action, Anticancer drugs, Antidiabetic drugs

## Abstract

Cancers are regarded as one of the main causes of death and result in high health burden worldwide. The management of cancer include chemotherapy, surgery and radiotherapy. The chemotherapy, which involves the use of chemical agents with cytotoxic actions is utilised as a single treatment or combined treatment. However, these managements of cancer such as chemotherapy poses some setbacks such as cytotoxicity on normal cells and the problem of anticancer drug resistance. Therefore, the use of other therapeutic agents such as antidiabetic drugs is one of the alternative interventions used in addressing some of the limitations in the use of anticancer agents. Antidiabetic drugs such as sulfonylureas, biguanides and thiazolidinediones showed beneficial and repurposing actions in the management of cancer, thus, the activities of these drugs against cancer is attributed to some of the metabolic links between the two disorders and these includes hyperglycaemia, hyperinsulinemia, inflammation, and oxidative stress as well as obesity. Furthermore, some studies showed that the use of antidiabetic drugs could serve as risk factors for the development of cancerous cells particularly pancreatic cancer. However, the beneficial role of these chemical agents overweighs their detrimental actions in cancer management. Hence, the present review indicates the metabolic links between cancer and diabetes and the mechanistic actions of antidiabetic drugs in the management of cancers.

## Introduction

Cancer is one of the major causes of mortality and is a significant health burden globally. In every year, the disorder leads to high cost of management for affected patients. Routine modalities for cancer management and treatment include radiotherapy, surgery and chemotherapy, used as a single treatment or combined therapy [1, 2]. Radiotherapy is usually applied for the treatment of localised cancers in addition to surgery, whereas latter is commonly used as the first-option intervention for the management of initial tumors. However, chemotherapy involves the utilisation of drugs with cytotoxic actions specifically to rapid multiplying cells. This method of intervention is vital in the treatment of several types of cancers but it is also associated with some limitations [2]. For instance, chemotherapeutic drugs may show cytotoxic actions on healthy multiplying cells, specifically in intestinal epithelial and bone marrow cells. Development of resistance against anticancer agents is among other setbacks in the use of chemotherapy. Hence, alternative interventions are needed to address these limitations. The use of other therapeutic agents is one of the strategies used in tackling some of the limitations presented by anticancer drugs [3, 4] such as multidrug resistance in cells with cancer and toxicity. Many classes of antidiabetic agents such as sulfonylureas (SUs), biguanides and thiazolidinediones (TZDs) were found to display useful actions in the management of cancer [5–7]. Moreover, several epidemiological works have revealed that there are metabolic links between certain cancers and diabetes, particularly type 2 diabetes which involves insulin resistance and pancreatic β-cells dysfunctions. In this regard, individuals with diabetes have high risk of bladder, colon, pancreas, ovary, breast and endometrium cancers [8, 9].

Although, the mechanisms linking these cancers and diabetes are still speculative, some of the risk factors for the two disorders may include hyperglycaemia, which could result in the formation of advanced glycated end products (AGEs) and oxidative stress; hyperinsulinemia, which normally occurs as a result of insulin resistance (impaired insulin function) or insulin from exogenous sources; inflammatory process as well as obesity [10]. Even though, diabetes is a risk factor for cancer and its prevalence, antidiabetic agents may show beneficial actions in the management of cancer [2]. In type 1 diabetes which is as a result of absolute insulin deficiency, treatment is based on exogenous insulin therapy, while in type 2 diabetes, treatment is usually based on antidiabetic drugs and lifestyle modifications. These drugs ameliorate the elevated level of blood glucose and other linked complications by stimulating the secretion of insulin by pancreatic β-cells, increasing insulin sensitivity to peripheral tissues, promoting the uptake of glucose into cells and reducing the reabsorption of glucose from renal tissues and intestine. Interestingly, these drugs were found to possess activity against cancer cells as they can inhibit the progression of the disease. Some studies showed that antidiabetic drugs can serve as a risk factor in cancer development or progression. Nevertheless, the action of these drugs against cancer cells outweighs their risk effects in the development or progression of this disease. Therefore, this work reveals some of the metabolic links between cancer and diabetes, the beneficial (repurposing) and risk effects in the use of antidiabetic drugs for cancer cells management and treatments.

## Diabetes and cancer

Most of the research conducted so far for delineating the linkage between diabetes and cancer have not made a demarcation between the two types of diabetes (type 1 and 2) that vary in several aspects like hormonal and metabolic characteristics. Due to prevalence of type 2 diabetes, the association between type 1 diabetes and cancer is not well described, as studies or epidemiological data on populations mostly comprises of subjects with type 2 diabetes, therefore, generalisation of findings might not be applicable to persons with type 1 diabetes. The observed differences in case of type 1 diabetes and cancer versus that between type 2 diabetes and cancer may be attributed to the deficiency of endogenous insulin secretion in the former and the coexistence of hyperglycaemia and hyperinsulinemia. Moreover, other risk factors, such as obesity, antidiabetic drug therapies such as intake of exogenous insulin [11] in type 1 diabetes, duration of the diabetes also influence the outcomes. However, due to number of limitations such as atypical samples, ambiguity in specifying type 1 diabetes, short perseverance or follow ups and improper assessment of the diabetes duration results into assorted findings for specific cancer type [12–15]. As carcinogenesis, is a multiplex process and involves multiple steps that can be affected or altered by diabetes via several mechanisms, this review delineates interlinking between these two diseases and the mechanistic actions of antidiabetic drugs in the management of cancers.

## Interlinks between diabetes and cancer: involving pathways

Although the linking of cancer and diabetes has been explored thoroughly, but some studies confounded that diabetes is associated with an increased risk of several types of cancer. Nevertheless, for the less common cancers, data are not sufficient to support this [16] and the reasons might be numerous, because more research is needed in this area. Since both diseases are complex and heterogeneous on their own and involve complex aetiology, therefore, interlinking links may be direct or indirect or through shared risk factors. In diabetics, cancer may be favoured by general mechanisms promoting cancer initiation or progression in any organ due to systemic alterations affecting all tissues and localised mechanisms affecting carcinogenesis of a particular organ [16]. Moreover, another complex issue is duration of diabetes and its multidrug therapy that also influences cancer risk [17]. A thorough understanding of whether diabetes influences cancer prognosis, high-quality databases and a prospective population-based studies are needed to compare and analyse the occurrence of specific cancers between subjects with or without diabetes and with the variations of insulin levels also. While analysing such factors, usual confounders must also be evaluated, like, physical activeness, body weight, age, comorbid conditions, medications and diet. A prospective study conferred that diabetes seemingly exert a remarkable influence downstream on the risk of mortality in people with cancer rather than on upstream risk of incident cancer as evident by the following outcomes [18]. Some likely features between diabetes and cancer are stated below:

- Diabetic adults are more likely to develop cancer than their non-diabetic counterparts, particularly pancreatic cancer.
- Diabetic adults are more likely to die of cancer than their non-diabetic counterparts.
- Diabetes was associated with greater cancer-specific case fatality for adults with cancer, particularly with colorectal cancer.
- In patients with cancer, individuals with diabetes had higher all-cause mortality than those without diabetes.
- In individuals with diabetes, the attributable fractions due to diabetes were larger for cancer case fatality and total mortality than for cancer incidence, with the exception of pancreatic cancer.

Plethora of cancer types have been documented to be remarkably linked to diabetes, though, interesting phenomenon of “reverse causality” is also there, in which cancer leads to diabetes onset as in the case of pancreatic cancer [15]. In contradiction to cancers of endometrium [19], colorectal [20], breast [21], bladder [22], kidney [23] and non-Hodgkin lymphoma [24], prostate cancer [25] is reported to be less likely in men with type 2 diabetes, which is seemingly attributed to the reduced levels of circulating testosterone in diabetic men. Prior studies elaborated that for some other cancers, there appears to be no consistent linkage between the two diseases for e.g. ovarian [26] and lung [27] cancer, as the studies conducted were limited. However, the advanced investigations revealed that diabetes is significantly associated with the lung cancer [28–30]. Increased mortality in lung cancer patients with diabetes mellitus has been extensively noticed in the recent years [31, 32]. An interesting meta-analysis came out that diabetes mellitus has no significant impact on the incidence of lung cancer in men but has a harmful effect on women [33]. It is to be noted that for some of the cancers, region/population specific association with diabetes might be there, that needed to be explored [34]. Analyses of type 1 diabetes cohort’s cases, as compared to general population, hinted an elevated risk in some cancers, but lacks consistency across all studies [35]. Moreover, the linkage between type 1 diabetes and the cancers associated with type 2 diabetes were also not found to be correlated [35]. It has been recommended that studies exploring the linkage between type 2 diabetes and cancer incidence should avoid overall cancer incidences, and instead focus on specific cancers so as to include the variations in peculiar patterns of site-specific cancer incidence involving biological, clinical or socioeconomic determinants [35].

### Hyperglycaemia

Impaired tolerance of glucose is usually found in cancer patients [36]. Therefore, an obvious question that arise about the type 2 diabetes and cancer linkage is the effect of high blood glucose level (hyperglycaemia). Research is still ambiguous about the role of higher circulating glucose in relation to malignant cell growth. However, it has been reported that hyperglycaemia increases production of free radicals and other reactive molecules that could results in oxidative damage to deoxyribonucleic acid (DNA) which is an initial step towards carcinogenesis, and eventually bring out mutations in tumor suppressor genes and proto-oncogenes [16]. Moreover, prolonged state of hyperglycaemia can be accessed via glycated haemoglobin level that indicates non-enzymatic and irreversible glycation of haemoglobin which could also results in oxidative stress and cancer. To support this, a study carried out on general Korean population showed that a 39.4% increase in glycated haemoglobin was related to a 3.03-fold increase in the risk of all types of cancer, suggesting the role of glycated haemoglobin as a causal risk factor for all cancer types [37]. However, data from large randomised controlled trials of intensified glycaemic control suggest that cancer risk is not reduced by improving glycaemic control in type 2 diabetes, therefore, do not support that hyperglycaemia is linked to increased cancer risk [38]. Additionally, because hyperglycaemia coexist with insulin resistance and obesity, its direct impact on cancer risk as an independent factor is difficult to analyse [16, 39]. Interestingly, hyperglycaemia in the absence of hyperinsulinemia has not been reported to provoke tumor growth in animal models, depicting the role of insulin receptor activation [17]. Hyperglycaemia also causes oxidative stress through inhibition of the antioxidant function of thioredoxin by Thioredoxin-interacting protein (TXNIP), an oxidative stress-responsive and glucose-inducible gene because it is a carbohydrate response element in its promoter and it is overexpressed in both diabetic animals and humans [40]. TXNIP inhibits thioredoxin, which is a ubiquitous oxidoreductase with antioxidant activity, by reversibly binding to its catalytic site, suggesting that TXNIP is an endogenous inhibitor of thioredoxin [41]. TXNIP has been reported to be strongly induced by glucose, and it is responsible for the impaired angiogenesis found in diabetes [42]. Furthermore, hyperglycaemia causes high rates of protein glycation and the subsequent formation of advanced glycation end products (AGEs) that contributes to long-term diabetic complications [43, 44]. Also, AGEs formation and their accumulation during hyperglycaemic conditions have been documented to be involved in carcinogenesis [45]. The effects of AGEs are direct to damaging of protein structures and extracellular matrix modification, and indirectly by binding the receptor for advanced glycation end products (RAGE). AGEs trigger oxidative stress in a diverse cell types by binding to RAGE, whereas oxidative stress itself induces AGE formation and increases the RAGE expression [46]. Furthermore, the binding of AGEs to their receptors (RAGE) leads to the induction of plethora of pathways. AGE-RAGE crosstalk also play a pivotal role in pancreatic cancer progression by inducing autocrine platelet-derived growth factor-B [47] and elicits thrombogenesis, angiogenesis, and vascular inflammation via Ras-extracellular signal regulated kinase-nuclear factor-kappa pathways [48]. Cancer cells are generally inclined towards anaerobic metabolism of glucose [49] for their energy needs and tumors are characterized by an increased glucose uptake as well as high rate of glycolysis to compensate for this inefficient energy supply. In consequence, enhanced glycolytic flux lead to an elevated level of glycation and thus increased formation of AGEs as a by-product. Thus, both diseases (cancer and diabetes) results into AGEs formation which in turn increase in AGE-RAGE-dependent stress response, leading to increased oxidative stress and chronic inflammation thus creating favourable environment for both, cancer and diabetes progression. High glucose under hyperglycemic conditions also modulates immune system functioning in a manner that glucose competitively disables the ascorbic acid transport into immune cells, [50] which is needed for effective phagocytosis, mitosis and for proper functioning of lymphocytes. Therefore, the immune response against cancerous cells lessens under hyperglycemic condition and it is evident that hyperglycaemia mediated high glucose supply to cancerous cells, facilitates anabolic metabolism and thus fuels its growth and thereby describes diabetes associated increased cancer risk. Wnt signaling, is another well-characterized cancer and diabetes associated pathway that links enhanced cancer risk with metabolic diseases such as hyperglycemia and obesity [51, 51]. Detail of this pathway has been explained in the sections below.

### Hyperinsulinemia

Role of insulin in carcinogenesis was primarily found in studies with experimental animals where hyperglycemic and insulin deficient models depicted less aggressive and lower number of tumors with slower cancer progression. Interestingly, insulin treatment reversed these effects, thus, these results are in accordance with the known mitogenic action of insulin [53].

The anabolic and anti-apoptotic actions of insulin promote tumor development in hyperinsulinemic subjects through binding of insulin to the insulin receptor (IR), the insulin like growth factor-insulin receptor (IGF-IR) or a hybrid receptor (IR-IGF-IR). In contrast to IR, which is highly expressed in adipose, muscle and kidney tissues, the IGF-IR is present greatly in all tissue types, signifying broad effects of insulin and IGF-1 [54]. Both receptors have been linked with tumor growth. Over-expression of IR was found in breast [54] and prostate[55] cancer cells and their higher expression has been reported to account for adverse prognosis [56]. Due to this, cancer cells can respond to insulin, especially in the conditions of obesity and diabetes where it is greatly expressed. The binding of insulin to its receptor stimulates downstream signaling insulin receptor substrates (IRS-1) or IRS-1/4 in which it further activates the RAS/RAF/MEK/ERK pathway, thus showing mitogenic effects whereas latter initiates the Akt/protein kinase B pathway and mediates anti-apoptotic effects of insulin [54]. Because of the homology, insulin and IGF-1 can interact either with IR or with IGF-IR [57]. Thus, insulin activates its metabolic actions by binding to the IR, and additionally stimulates growth and differentiation by binding to the IGF-IR. Additionally, a third receptor can mediate insulin and IGF-1 actions. Due to high homology, IR and IGF-IR can form a hybrid receptor [58] if they are co-expressed in the same tissue and their over-expression in malignant breast and thyroid tissue via heterodimerization has been reported [54]. Moreover, it binds IGF-1 with a much higher affinity than insulin and induces the cell proliferative activity.

Interestingly, insulin/IGF may also stimulate normal cells that could assist in cancer progression, as hyperglycaemia allows IGF-1 to stimulate vascular smooth muscle cell proliferation and migration [59] leading to abnormal vasculature growth, a hallmark of cancer. Hyperinsulinemia could indirectly promote carcinogenesis via IGF-1 [60]. Insulin reduces the hepatic production of IGF binding protein 1 and 2 IGFBP-1/2) (which normally bind to and inhibit the action of IGF-1), that results into increased levels of circulating free IGF-1 [61, 62]. It is pertinent to mention that IGF-1, being a more potent mitogen and anti-apoptosis inducer than insulin, [63] could act as a growth stimulator in pre/neoplastic cells that express insulin, IGF-1, and hybrid receptors [64]. Moreover, hyperinsulinemic condition also leads to the release of the proinflammatory cytokines which will be discussed under separate section of the manuscript.

### Overweight and obesity

Overweight and obesity in combination with lack of physical activity are well-known risk factors for the development of cardiovascular ailments and diabetes, but surprisingly less discussed or explored risk factors especially for adult cancers [65]. Several biological mechanisms link obesity with tumorigenesis. One of the most common mechanism accounts for the relationship between the two aforementioned conditions are some shared risk factors such as hyperinsulinemia, antihyperglycemic medications and chronic inflammation [43]. Moreover, an increment in circulating sex steroids due to overweight is also a well-known factor involved in carcinogenesis. Frequently reported obesity- and diabetes-related cancers are colorectal, endometrial and postmenopausal breast cancers [66]. Obesity is the obvious outcome of chronic excess energy intake and it is the strongest casual factor of hyperinsulinemia and insulin resistance. In particular, visceral obesity leads to metabolic abnormality and enhanced release of free fatty acids, resistin, tumor necrosis factor alpha (TNF-α), whereas reduced release of adiponectin into the circulation eventually results in the development of insulin resistance and chronic hyperinsulinemia. Insulin resistance is a feature that is very usually linked with obese people and it results in high level of circulating insulin, a well-established risk factor for cancer and which is also accompanied by marked changes in the levels of inflammatory markers [67].

Obesity-linked insulin resistance and hyperinsulinemia results in elevated level of unbound IGF-1 protein [68] in blood, with triggering of the insulin and IGF-1 receptors signal transduction pathways that eventually promotes tumor growth [69]. As discussed above, mean concentrations of IGF binding proteins (IGFBPs) in obese persons are lower as compared to non-obese. This inverse relationships between the concentrations of these factors and insulin is due to the direct suppression of IGFBPs by insulin [70]. Several studies have linked elevated levels of serum C-peptide (a stable marker of insulin secretion that is co-secreted with insulin) with enhanced risk of post-menopausal endometrial cancer, colorectal cancer and breast cancer, but not pre-menopausal, ovarian, breast and prostate cancers. This is consistent with remarks that the former cancers are linked primarily with obesity [62]. Interestingly, some studies on prostate cancer have depicted ambiguous correlation between circulating C-peptide and either prostate cancer risk, or mortality, or the risk of developing aggressive stage [71, 72]. Although, chances of mortality from prostate cancer in obese men with enhanced C-peptide levels than men with normal C-peptide levels were approximately four times [73]. Adipose tissue is an organ that accounts for the generation of adipokines and various enzymes that are impaired in obesity and type 2 diabetes and possibly contribute to carcinogenesis. Obesity and type 2 diabetes induced alterations in adipose tissue may cause the liberation of factors into the circulation that promote tumor growth. Moreover, adipose tissue may have an important impact on cancer cells residing in the neighboring tumor *milieu*. Some of the factors released via adipose tissue and adipose tissue macrophages that are related to cancer includes adiponectin, leptin, lipocalin 2, resistin, nicotinamide phosphoribosyltransferase, interleukin-6 (IL-6) and TNF-α [74].

Additionally, in corroboration with systemic endocrine modulations i.e. hyperinsulinemia, increased estrogen levels in obesity may be a crucial factor for tumor development. Obesity mediated carcinogenesis occurs via imbalance of adipocytokines that includes enhanced production of leptin (oncogenic adipokine) with lessened release of adiponectin. Interestingly, adiponectin is one of the most important adipocytokines secreted by adipocytes that is inversely associated with visceral adiposity and body fat mass and it can trigger plethora of signaling pathways like mitogen-activated protein kinase (MAPK), adenosine monophosphate-activated protein kinase (AMPK) and phosphoinositide 3-kinase (PI3K)/Akt resulting to inhibition of tumor formation induction. Moreover, it also triggers tumor suppressor gene liver kinase B1, leading to inhibition of cell invasion, migration, metastasis, but it induces cytotoxic autophagy. Various in vitro and in vivo studies have documented its role in induction of growth arrest and apoptosis as well as inhibition of angiogenesis [75]. Leptin, a key regulator between energy metabolism and immune system, is also responsible in part for the obesity linked inflammatory state. Leptin signaling influences numerous molecules involved in inflammation, angiogenesis, proliferation, adhesion, invasion and migration, especially in breast carcinogenesis [76]. Increased risk of colon cancer [77] and breast cancer [78] have been reported to link with high leptin levels. Moreover, postmenopausal women with the highest waist circumference and leptin concentration are documented to have maximum risk of breast cancer [78]. Although, some researchers differ with a fact that leptin plays role in the breast cancer etiology [79]. However, an enhanced leptin receptor expression has been found in various cancer types [67]. Interestingly, it has been reported that cells can have stress in a condition of “nutrient excess” associated with obesity as well, where reactive oxygen species (ROS) generation exceeds what is required for normal physiological responses. Enhanced ROS, produced largely from the mitochondria, may promote cancer by increasing DNA mutation, by regulating signaling and transcription, and by promoting inflammation [80]. Additionally, the obesity induced inflammatory effects may promote cancer cell survival and further progression via enhanced generation of systemic inflammatory responses such as interleukin (IL)-1, IL-6, tumor necrosis factor (TNF)-α, cyclooxygenase-2, plasminogen activator inhibitor-1, fibrinogen, C-reactive protein and others [81, 82]. Besides, inflammatory processes in breast cancer also,  activation of nuclear factor-kappa B (NF-Κb), a transcription factor is associated to inflammation and the development and progression of tumors [83]. Another adipokine found in an enhanced concentration in adipose tissue of obese persons is ceruloplasmin which is synthesized and released at higher rates as compared to control subjects [84]. Ceruloplasmin is involved in angiogenesis, and its higher concentration possibly facilitate or promote carcinogenesis in obese subjects [67].

### Impaired cell signaling cascades (Wnt/β-catenin and mammalian target of rapamycin pathways)

#### Wnt/β-catenin signaling pathway

Wnt/β-catenin signaling pathway is a prime pathway as it governs cell proliferation and physiological processes including embryonic development, cell migration/polarization, maintenance, expansion and epithelial-mesenchymal transition of stem cells [85, 86]. Wnt/β-catenin signaling pathway regulates and maintains these processes through the transcriptional control of the involved genes. Any alteration and mutation in certain components of this pathway are related with human birth defects, occurrence of different types of cancer (via affecting the behaviour of cancer stem cells) such as hepatocarcinoma, colon cancer, leukemia and other metabolic diseases such as type 2 diabetes [87–90]. There are two main categories of Wnt pathways, that is, the most studied canonical Wnt signaling pathway (β-catenin-dependent) and β-catenin-independent non-canonical signaling pathway. Generally, canonical Wnt cascade function by controlling the amount of the transcriptional co-activator β-catenin that further regulates self-renewal, proliferation, or differentiation of progenitor cells along with the developmental gene expression programs [86, 91]. Moreover, non-canonical pathway is involved in the maintenance of stem cells, cell movement, or inhibition of the canonical pathway [90].

Canonical Wnt/β-catenin pathway is one of the most prominent link between cancer and diabetes. Basically, hyperglycaemia promotes cancer associated Wnt signaling as it allows nuclear retention and accumulation of β-catenin which consequently permit incessant expression of Wnt/β-catenin-dependent target genes that promotes cell proliferation, survival and senescence bypass [52, 92]. As increased glucose uptake is a key marker of malignant cells which may ensure enhanced Wnt signaling for continuous cell proliferation [92, 93]. Generally, high glucose level promotes lymphoid enhancer factor/β-catenin complex formation which results in increased p300 acetyl transferase activity and decreased sirtuin-1 deacetylase (SIRT1 deacetylase) activity that subsequently increases β-catenin acetylation at lysine 354 position. This acetylation β-catenin at lysine 354 is necessary for nuclear accumulation and transcriptional activation of Wnt-target genes [92]. Therefore, it would not be wrong to suggest that hyperglycaemia provide a boost in cancer associated Wnt/β-catenin pathway and elucidate the raised frequency of cancer associated with obesity and diabetes. Wnt/β-catenin signaling pathway has a strict and efficient regulation mediated by positive and negative feedback regulators. However, any mutation and deregulation in the component of this pathway may lead to more critical conditions. It has been submitted that the loss in scaffolding function of adenomatous polyposis coli, the multifunctional tumor suppressor, results in constitutive activation of Wnt/ β-catenin signaling and subsequent formation of pre-malignant intestinal polyps predominantly in colorectal carcinomas [94]. Generally, frameshift and nonsense mutations have been noticed in adenomatous polyposis coli that leads to protein truncation. Mutation in Axin has also been confirmed in colorectal and hepatocellular carcinomas, whereas point mutation that makes β-catenin defiant for phosphorylation generates various types of tumors [94, 95].

There are two cell-surface, single-pass transmembrane, homologous E3 ligases, i.e. E3 ubiquitin ligase zinc and ring finger 3 (ZNRF3) and ring finger 43 (RNF43). Both are encoded by Wnt target genes and form a negative feedback loop [96, 97]. RNF43 and ZNRF3 are powerful down-regulators of the Wnt/β catenin pathway. They are involved in ubiquitination and lysosomal degradation of Fzd and LRP5/6. Loss-of-function mutation of RNF43 and ZNRF3 leads to the hyper-responsiveness of Wnt signals and deregulations of R-spondin/ZNRF3/RNF43 feedback loops which is marked by various forms of cancers [98]. R-spondin is a secreted protein that prevents the LRP5/6 internalization and acts as an activator of Wnt/β-catenin signaling. Colorectal carcinoma, tongue squamous cell carcinoma and endometrial cancer are prime consequence of over-expression of R-spondin [99, 100]. Mutation in RNF43 is more frequent than ZNRF3 and its associated repercussions are pancreatic tumors called intraductal papillary mucinous neoplasm and mucinous cystic neoplasm, colorectal adenocarcinomas, pancreatic cancer, gastric cancer, mucinous ovarian carcinoma, and endometrial carcinomas. Although adrenocortical carcinoma is the only cancer type which has been documented owing to the mutation in ZNRF3 [96, 101].

#### Mammalian target of rapamycin (mTOR)

mTOR is a decisive signaling cascade that efficiently integrates extracellular and intracellular signals to control cell survival, growth and proliferation along with metabolism. mTOR signaling pathway have been linked to insulin resistance, tumor formation and angiogenesis, T lymphocyte activation and adipogenesis [102]. Cancer and type 2 diabetes are frequently noticed repercussions owing to the deregulation of this pathway [103]. Basically, mTOR is a large (289-kDa) serine-threonine kinase, an evolutionary conserved protein having association with phosphoinositide 3-kinase (PI3K) related family [104]. mTOR exists in two functionally distinct multiprotein complex in cells i.e., rapamycin sensitive mTOR complex 1 (mTORC1) and rapamycin insensitive mTOR complex 2 (mTOR2). mTORC1 promotes several anabolic (biosynthesis of lipids and organelles) processes and restrict catabolic processes (autophagy), thus, controls cell growth and proliferation [105]. It has been found that inhibition of mTORC1 leads to arrest of G1 phase of cell cycle with reduced cell proliferation rate [106]. However, mTORC2 is involved in cell proliferation/survival [107] and activates AKT, protein kinase Cα and serum/glucocorticoid-regulated kinase 1 by phosphorylating them [108, 109]. Activated AKT through phosphorylation and inhibition of several key substrates, such as FoxO1/3a transcription factors, glycogen synthase kinase-3 (metabolic regulator) induces cell survival, proliferation, and growth [110]. Additionally, protein kinase Cα govern cytoskeletal organization whereas serum/glucocorticoid-regulated kinase 1 regulates ion transport as well as cell survival [111].

The molecular mechanism and regulation of mTORC1 has been extensively studied in comparison to mTORC2 [112]. Insulin and IGF induce mTORC1 activation mainly through the PI3K/AKT signaling pathway. AKT indirectly activates mTORC1 by phosphorylation-based inhibition of tuberous sclerosis complex 1 and 2 (TSC 1/2), a GTPase-activating protein and acts on G-protein Ras homolog enriched in brain that activates mTORC1 and subsequently promotes its further action such as cell growth. Whereas, various growth factors also regulate mTORC2 through PI3K which subsequently activates AKT pathway [105]. With the significant advancement of understanding the functioning and regulation of mTOR, it is quite clear that these proteins are critically involved in the onset and progression of diabetes and cancer. mTORC1 signaling regulates functioning and cell mass of pancreatic β cell and influence glucose homeostasis [110]. The constitutive activation of mTORC1 in pancreatic β cell has a biphasic effect on pancreatic β cell function which has been conferred through experiment on mice. Increased pancreatic β cell mass, hyperinsulinemia and improved glucose tolerance has been noticed in young β cell-specific TSC2 knockout (b-TSC2KO) mice whereas reverse conditions have been noticed in older b-TSC2KO mice with reduced β cell mass, hypoinsulinemia, and hyperglycaemia [105, 110]. Hence, it is clear that enhanced pancreatic β cell activity is good for glucose tolerance during initial stages, however, there was fast decline in pancreatic β cell function over time. This biphasic condition is similar as in diet-induced (type 2) diabetes progression in which increased glycaemic load causes β-cell hypertrophy and proliferation to increase production and secretion of insulin (condition called β-cell compensation) and eventually get exhausted due to constant pressure. In β-cells, decreased AKT activity and activation of FoxO1 is attributed to loss of mTORC2 that resulted into glucose intolerance and mild hyperglycemia as the mass of β cell get decreased consequently to decline in production and secretion of insulin [105].

Aberrant activation of mTORC1 is proved as hallmarks in certain type of cancer [113]. Tumor development and angiogenesis have been noticed in many in vitro cell lines and in vivo murine xenograft models because of certain oncogene stimulation or failure of tumor suppressors genes that results into anomalous activation of mTOR pathway [105]. Mutation has been one of the prime cause of cancer and mutations in upstream and downstream components of mTORC1 may leads to aberrant mTOR signaling and resulting into cancerous conditions. Mutation in six different regions of the c-terminal region of mTOR, the core gene of mTOR signaling is associated with constitutive over-activation of mTOR signaling [114]. In addition to it, mutation in upstream genes (oncogenes and tumor suppressor genes) is also responsible for over-activation of mTOR. As mTOR is key element of PI3K/AKT/mTOR signaling pathway and functioning, its regulation is influenced due to hyperactivation of growth factor receptors including IGFR, human epidermal growth factor receptor 2 and mutation in PI3k and AKT [104, 105]. As mTOR1 belong to Ras/MAPK and PI3K/AKT signaling pathways, any alteration in the components may results into critical conditions leading to cancer. Gain-of-function mutation in Ras, Raf, PI3K and AKT (activating mutation) oncogenes and loss-of-function mutations in the TSC, PTEN (phosphatase and tensin homolog) and neurofibromatosis-related protein-1 may lead to constitutive activation of mTORC1 that results in anabolic processes driving tumor cell growth and proliferation [107]. Variety of tumors arise due to mutation in the catalytic and regulatory subunits of PI3K, moreover, increased activity of PI3K has been noticed in Ras mutation [115]. Events such as activation mutation in PIK3CA encodes p110α subunit of PI3K, deletion of PTEN, over-expression of AKT itself and epidermal growth factor receptor (EGFR) is associated with aberrant activation of AKT which is an oncogenic phenomenon [116]. TSC1 and TSC2 complex negatively regulate the activity of mTOR1 and connection between TSC and the mTORC1 pathway revealed very first molecular link between mTOR and cancer, although, AKT based phosphorylation and inhibition of TSC2 is the clearest link between mTORC1 and dysregulated pathway of cancer [116]. Inhibition of TSC1 and TSC2 leads to Tuberous sclerosis and benign tumorigenesis. Although, mutation in TSC1/TSC2 and mTOR is less common in comparisons to the higher upstream components of the signaling pathway [104]. Colorectal, breast endometrial, prostate cancers, glioblastoma, melanoma and lymphoid malignancy were observed as a result of mutation or deletion of PTEN genes, which is considered as second most mutated gene in case of human cancer after p53 [117–119]. The activities of PTEN get hampered by mutation, methylation, protein instability and intracellular localization [104, 119]. The downstream effectors of mTOR such as, S6 Kinase 1, 4E-binding protein 1, and eukaryotic translation initiation factor 4E (eIF4E) can cause tumorigenesis. Overexpression of oncogene eIF4E has been noticed in several human cancers with poor prognosis. The eIF4E is involved in translation of pro-oncogenic proteins coding mRNA that subsequently influence cell proliferation and tumorigenesis [120]. More phosphorylation and dysregulated expression of 4E-binding proteins resulted in poor prognosis in cancer patient. Moreover, overexpression of S6 Kinase 1 has been noticed in lung and ovary cancers along with brain tumor [120]. In summary, mTOR plays major role in cell growth and proliferation, hence, it is a principal target in cancer therapy.

### Role of severe inflammation

Diabetes impacts a huge risk for liver and pancreatic cancers as both organs are exposed to high concentration of endogenously produced insulin. Hyperinsulinemia and hyperglycaemia have role in stimulating the growth of cancerous cells and cause higher risk for malignant transformation [121]. Poorly controlled diabetes is characterized by long term proinflammatory conditions that greatly induce IL-6, TNF-α and other chronic inflammation markers [122]. Diabetes-related factors, steatosis, non-alcoholic fatty liver disease, hepatitis B virus or hepatitis C virus infection, cirrhosis, aflatoxins exposure and excessive alcohol consumption may lead to liver injury and consequently severe liver inflammation [123]. Persistent inflammation is associated with genetic instability and enhances susceptibility to cancers [9] of which liver and pancreas show the highest increase in risk. Although liver has compensatory regeneration mechanism, but excessive compensatory proliferation can generate consecutive pathological changes and enhance the risk of genetic mutation in hepatocytes that further promotes hepatocarcinogenesis.

Hepatocellular carcinoma (HCC) is the most extensively investigated cancer reported from inflammatory and hepatic injury cases [124]. Every year, about one million HCC cases are diagnosed with substantially identical death rate. Globally, HCC is recognized as fifth most common malignancy and the third leading cause of cancer-related deaths [125]. Whereas, pancreatic ductal adenocarcinoma is the most devastating cancer, and it was ranked at fourth position for the death rate globally [1]. There are numerous kinds of cytokines, chemokines, transcription factors and proteins which belong to inflammatory signaling pathways implicated in hepatocarcinogenesis and pancreatic ductal adenocarcinoma. These complex signaling molecules and pathways are interconnected with extensive crosstalk [123, 126, 127]. Inflammatory cytokines such as TNF-α, IL-1α, IL-1β, IL-6, IL-8 play a key role in chronic hepatic inflammation as its up-regulation has been noticed in liver inflammation [128, 129]. Hepatocyte expresses a receptor called glycoprotein 130 (gp 130) for IL-6 and binding of IL-6 to its receptor leads to phosphorylation of gp130 by Janus kinase (JAK). This event induces multiple signaling pathways including JAK/signal transducer and activator of transcription 3 (STAT3), PI3K/Akt and Ras/Raf/MAPK pathways that are crucial for hepatic regeneration by blocking and reducing apoptotic cascade and oxidative injury [124, 130]. Activated kupffer cells also produce IL-6 during chronic hepatitis that further boost up the local inflammatory responses and stimulate liver for compensatory hepatocyte proliferation, consequently, this leads to neoplastic transformation of hepatocytes [125]. Furthermore, IL-6 promotes glycolysis and IL-6-activated STAT3 induces the expression of glycolytic enzymes such as hexokinase-2 and 6-phosphofructo-2-kinase/fructose-2,6-bisphosphatase-3, hence, this shows the association between oncogenesis and inflammation [123, 131]. IL-6 and TNF-α are cytokines that links the obesity and liver cancer through chronic inflammation and contribute for development of chronic low-grade systemic inflammation [132–134]. Moreover, in type 2 diabetes, concentration of IL-6 was noticed to be considerably high which is also linked to HCC [134–136].

In pancreatic cancer, IL-6 induces the STAT3 signaling pathway and thus cancer cell proliferation. Moreover, it promotes the release of T helper 2 type cytokines along with extracellular signal regulated kinase 2 (ERK2) signaling pathway [126]. Hence, it can be sum up that IL-6 generates a tumor environment by inducing the genes involved in cell proliferation. Nevertheless, other cytokines that proficiently engaged in pancreatic cancer cell proliferation are IL-4 and IL-8. Ablation and inhibition of IL-4 and IL-8 in different cell lines showed reduced cell growth that confirm their functionality in cancer [137, 138]. Other pro-inflammatory cytokines are IL-1α and IL-1β which play role in invasion, metastasis and angiogenesis. Role of IL-1β has also been established in hepatic inflammation induction by the production of C-reactive protein, a marker of infection and inflammation [125, 139]. To sum up, the multiple roles of these cytokines in both the diseases provides a strong interlinking that may answer the questions related to their co-occurrence.

NF-κB, a transcription factor, is another important factor that is involved in the regulation of inflammatory signaling pathway [140–142]. In many solid tumors, activation of NF-κB has been noticed, however, no oncogenic mutations responsible for activation of NF-κB in carcinomas have been identified. Activation of NF-κB in such cancers owes to inflammation or inflammatory microenvironment formed during malignant progression [141]. Study conducted on mouse models has confirmed that IκB kinase/NF-κB signaling play sharp contrast roles as it is involved in tumor-suppression and tumor development role in mouse hepatocyte and HCC mouse model [143]. Moreover, it has been noticed that in case of pancreatitis, NF-κB pathway is activated in early stages and cause pro-inflammatory response through the activation of anti-apoptotic and inflammatory genes [126]. The level of NF-κB correlates with the severity of acute pancreatitis which in turn induces the release of inflammatory cytokines and these cytokines promote tumorigenesis.

Interestingly, NF- κB has been also implicated in the progression of type II Diabetes [144] involving modified levels of specific chemokines and cytokines, alteration in levels and activation state of different leukocyte populaces etc. that eventually strengthens insulin resistance [136]. Thus it can be hypothesized that since NF- κB induced pro-inflammatory states may provide a potential linkage between diabetes as well as cancer [145] that may be of immense potential in targeting drugs against these diseases.

Previous studies revealed that STAT proteins are involved in cytokinin signaling pathway that regulates cell growth and differentiation [146]. Among all the members of STAT family, STAT3 fascinated researchers because of its involvement in proinflammatory cytokines signaling and along with oncogenic signal cascading [147]. STAT3 get activated by a range of cytokines, and growth factors. Activated STAT3 was found in more than 60% of human HCC samples, thus, phosphorylated STAT3 level is linked with the aggressiveness of the tumors [142]. STAT3 governs an early event that is acinar-to-ductal metaplasia during pathogenesis of pancreatic cancer [148]. Moreover, STAT3 has role in progression of pancreatic cancer precursor lesions, cell proliferation and metaplasia associated inflammation that subsequently leads to pancreatic ductal adenocarcinoma initiation [126, 149]. STAT3 may be a safe target for cancer therapeutics as STAT3 deletion does not affect the viability of differentiated cells, but proficiently blocks the cell proliferation [142]. On the other hand, constitutive STAT3 phosphorylation has also been reported to contribute to skeletal muscle insulin resistance in type 2 diabetes [150]. Therefore, involvement of STAT3 needs to be explored at the molecular level in the subjects suffering with both, cancer and diabetes.

EGFR, a transmembrane glycoprotein of the tyrosine kinase family, encoded by proto-oncogenes is one of the links between liver inflammation and HCC [125, 151]. It has been noticed that over-expression of EGFR is related to the severity of liver and pancreatic tumors and elevated death rate [152, 153]. The experiments conducted on human cell lines and different animal models concluded that continuous activation of EGFR signaling is a prime marker of HCC leading to proliferation, resistance to apoptosis and invasive behaviour of HCC cells [151]. Also, progression of pancreatic cancer is linked with up-regulation of EGFR [154] and the glycoprotein over-expression has been noticed in human pancreatic cancer, a cell line that is up to 85% of ductal adenocarcinomas whereas percentage of silent mutation was 81% [154, 155]. Although, overexpression of EGFR in pancreatic cancer was established through numerous evidence but data on the prognostic significance of EGFR expression is still lacking [153]. Apart from EGFR, human chemokines also has association with inflammation and tumorigenesis [156]. With the help of chemokine, tumor cells induce the growth of tumor and the recruitment of inflammatory cells. Additionally, chemokine also contribute in HCC progression, growth, and metastasis, as well as immune response to HCC [125].

EGFR plays a significant role in the progression of diabetic kidney disease (DKD) evident by significantly increased level of phosphorylated EGFR levels in animal models of diabetes mellitus and in cultured cells treated with high glucose [157, 158]. In accordance to these results others also reported that inhibition of EGFR slowed the progression of DKD, leading to the improvement in condition of proteinuria and morphological changes [159]. Besides direct activation by its ligands, the process of EGFR transactivation also occurs via second messengers such as ROS, TGF-β and protein kinase C (PKC). EGFR has been reported to contribute to DKD via inflammatory responses as its inhibition decreases oxidative stress, renal T-cell infiltration and islet macrophage infiltration in diabetic glomeruli and the interstitium [159, 160]. Recently, it has been found that targeting EGFR might also hold a therapeutic potential for Diabetic kidney disease [161].

### Role of oxidative stress

Oxidative stress is a physiological state in which generation of ROS and free radicals overwhelms the body’s antioxidant system. Mostly, ROS and free radicals are produced via mitochondrial respiratory chain and other endogenous metabolic reactions [162]. They are also produced during disease conditions such as diabetes and cancer. They are involved in various signaling pathways, defense against microbial pathogens under low concentration but their imbalance cause damage to important biomolecules (lipids, carbohydrates, proteins and DNA) and cell [162, 163]. The consequences of ROS mediated DNA damage includes arrest or induction/replication errors, or genomic instability, and all these conditions induce carcinogenesis [164].

Oxidative stress is allied with several pathogenesis such as neurodegenerative disorder, hypertension, aging, inflammation, apoptosis, cardiovascular disease, diabetes, and cancer [164, 165]. It has been noticed that oxidative stress is the “final common pathway”, through which risk factors of several diseases arises and diabetes and cancer are two of them. Diabetes is characterized by hyperglycemia and this condition triggers several metabolic signaling pathways such as cytokines secretion, inflammation, cell death and subsequent diabetic vascular complications [166]. Hyperglycemia induced diabetic complications come into view owing to ROS, which induces oxidative stress leading to cell-death [167]. In diabetes or insulin resistance, there is higher oxidative glucose metabolism which itself enhances the mitochondrial production of ROS such as O_2_^•^ ‾ which is subsequently converted into OH^•^ and H_2_O_2_ [168]. On the other hand, in diabetic condition, over-activation of uncoupling proteins of inner membrane of mitochondria leads to superoxides formation. Nicotinamide adenine dinucleotide (NAD) oxidase is also responsible for hyperglycemia-induced oxidative stress in diabetes [168] and it catalyzes O_2_^•^ ‾ formation through the reduction of an electron on the electron transport chain in mitochondria. This activity is also carried out by xanthine oxidase [169]. Rise in ROS in hyperglycemic condition may be due to the involvement of different pathways including (a) increased flux of glucose through the polyol pathway (b) elevated level of AGEs synthesis and its receptor activation (c) protein kinase C isoforms (α, β, δ) activation (d) hexosamine pathway over-activation (e) and reduced antioxidant defenses [170]. Evidences revealed that oxidative stress-responsive genes such as thioreodoxin-interacting protein are sensitive to hyperglycemia, hence, increase oxidative stress may affect their functionality [16]. It has also been submitted that pancreatic β cells express antioxidant enzymes such as catalase, superoxide dismutase and glutathione peroxidase in very less amount, hence, the susceptibility of pancreatic β cells increases for oxidative stress that leads to the development of diabetic complications [171]. Auto-oxidation of glucose and shifts in redox balances with reduced tissue concentration of vitamin E is often noticed as a source of oxidative stress in diabetes. Malfunctioning of mitochondria in diabetes also reduces the energy supply for most vital energy dependent DNA repair process and increases ROS production, [16] hence, promote cancerous conditions.

Prolonged oxidative stress induces chronic inflammation that is responsible for diabetic complications and this can induce the transformation of normal cell into tumor cells and consequently several types of cancers. During inflammation, accumulation of cells such as polymorphonuclear neutrophils and mast cells at injury site leads to respiratory burst and release several ROS [169, 172]. The release of soluble mediators (cytokines and chemokines) by inflammatory cells leads to recruitment of more polymorphonuclear neutrophils (PMNs) and mast cells at site of injury, hence, more ROS are produced. The released mediators produce immediate cellular stress response by inducing transcription factors including NF-κB, hypoxia-inducible factor-1α, NF-E2 related factor-2, STAT3, activator protein-1 and nuclear factor of activated T cells [173]. Dysregulation in the expression of microRNAs and inflammatory cytokines such as TNF-α, IL-1 and IL-6 can be noticed in inflammation induced by oxidative stress. Furthermore, TNF-α is correlated to insulin resistance, this molecule (TNF-α) and ROS can activate the transcription factor of NF-κB, which leads to the induction of genes responsible for carcinogenesis, cell proliferation and apoptosis [164]. As a result of this, chronic inflammation is accountable for angiogenesis as generated ROS enhances the rate of expression of transcription factors for c-Fos and c-Jun which is responsible for neoplastic transformation and cancer angiogenesis [174, 175]. In addition, extended oxidative stress and inflammation cause injury to healthy cells and may produce carcinogenic effect. In the past few decades, increasing evidence have proved the association between diabetes and cancer through oxidative stress. As diabetes is linked to elevated free reactive radical levels and declined antioxidant state, it enhances the risk of DNA damage and other impairments. DNA damage through oxidative stress induces transcriptional arrest, replication errors, genomic instability and all these conditions collectively lead to carcinogenesis [164]. Hence, association between diabetes and cancer with increasing prevalence of diabetes among population is challenging for researchers to investigate new remarkable preventive measures that effectively decrease morbidity and mortality risk.

### Role of reproductive hormones

The change in the bioavailability of reproductive hormones in diabetic human male and female generates carcinogenic environment. Varying bioavailability of ovarian steroid hormone, that is, elevated level of estrogen, androgen and the declining level of progesterone in diabetic female potentially arouse breast, endometrium, and ovaries malignancy [122]. In circulatory system, sex steroid hormones are present in bounded form with a glycoprotein named sex hormone-binding globulin and it is synthesized in the liver. The bioavailability of these hormones firmly depends on sex hormone-binding globulin and it has been reported that hyperinsulinemia is responsible for reduction of concentration of the circulating sex hormone-binding globulin, therefore, it enhances the level of bioactive estrogen in diabetic female [16]. Also, hyperinsulinemia promotes androgen synthesis in the ovarian stroma. Normally, estrogen is involved in the proliferation of endometrium during menstrual cycle and also responsible for ductal elongation in mammary gland development during puberty [122, 176]. Based on these, dysregulation in the functionality of estrogen may generates conditions that could result to expansion of the cells. Binding of estrogen-to-estrogen receptors subtypes estrogen receptor alpha and beta (a family of ligand-activated nuclear receptors) bring the physiological changes. Binding of estrogen to estrogen receptor alpha in cancerous cells elicit the growth and proliferation of cancerous cells by subsequent activation of PI3-K and MAPK pathways [177]. Predominant expression of estrogen receptor alpha has been noticed in reproductive organs, kidney, bone, liver and white adipose tissue but major availability of estrogen receptor beta can be seen in ovary, prostate, uterus, bladder and central nervous system [178]. The risk of cancer increases in diabetic patient as target tissues of ovarian steroid hormones have higher concentration of IGF-1 and increased expression of IGF1R, IRS-1 and IRS-2. Activated IR and IGF1R have concerned with estrogen receptor alpha phosphorylation that subsequently promotes estrogen receptor alpha signaling.

In aggressive tumor, increased expression of estrogen receptor alpha has been noticed whereas the expression of estrogen receptor beta was completely absent. Approximately in 70% of ovarian cancer patients, continuous expression of estrogen receptor alpha occurs, and this receptor can be targeted for the efficient treatment of ovarian cancer [178]. Diabetic and obese females are likely to have increased risk of breast cancer. The reproductive hormones act as one of the governing factors for breast cancer and estrogen plays significant functions in normal mammary gland development whereas it also promotes the breast cancer growth in impaired conditions. It has been confirmed that there is rise of about two-fold in risk rate of postmenopausal breast cancer due to the elevated level of endogenous estrogen [179]. Obese and insulin resistant females have lower level of sex hormone-binding globulin and elevated level of estrogen because of increased activity of aromatase in the adipose tissue that results in peripheral conversion of androstenedione and testosterone to estrone and estradiol respectively [180]. Owing to this combined effect, there is an increased bioavailability of estrogen in the circulatory system and the binding of sex steroid hormones with their receptors can produce different effects that depend on tissue type but in tissues like breast, epithelium and endometrium, it triggers cell proliferation and inhibition of apoptosis. During cancer development, insulin, IGFs, and ovarian steroid hormones (estrogen and progesterone) can act synergistically [122]. A study [104], revealed that estrogen interact with insulin synergistically to promote type 1 endometrial cancer, however, the specific effects and underlying mechanism(s) of synergy remains unclear. Moreover, conflicting association has been noticed between diabetes and the risk of prostate cancer among different population. Studies conducted to investigate the relationship between diabetes and prostate cancer from United States demonstrated an inverse relationship whereas a significant increased risk for prostate cancer was observed in Asian population [9, 181]. The protective effect of diabetes in prostate cancer may be linked to the lower level of testosterone in diabetic men. Insulin positively influence the growth of both normal and cancerous prostate cells and reduced level of insulin in diabetic male may not affect the growth of cell [182]. Consistently, elevated risk of prostate cancer in certain populations may be associated with the difference in the distribution of prostate cancer risk associated genotypes i.e. AR, SRD5A2 and VDR [181]. Although, increased risk is also linked to prostate cancer diagnosis as diabetic men has lower levels of testosterone and prostate-specific antigen that reduces the chance of prostate-specific antigen screening in identifying early prostate cancer [181, 183, 184]. It was noticed that relationship between diabetes, sex-hormone levels and prostate cancer is complex, more efficient communication strategies are required between clinicians and individuals with diabetes and prostate cancer.

## Antidiabetic drugs with anticancer actions

Disorders like diabetes and obesity characterized by insulin resistance [185] pose a high risk for the development of different types of tumors such as breast, liver, colon, endometrial and pancreatic cancers [186–188]. During the last two decades, several clinical studies have claimed that few of the antidiabetic drugs can protect patients against several types of cancer [189]. Despite the fact that many antidiabetic medications are currently available in the market, some antidiabetic agents such as biguanides, SUs and TZDs have been reported to exert an antiproliferative effect on many cancer cell types. This phenomenon has triggered an intense research in this area in the recent years [190–192].

### Sulfonylureas

**Fig. 1:**
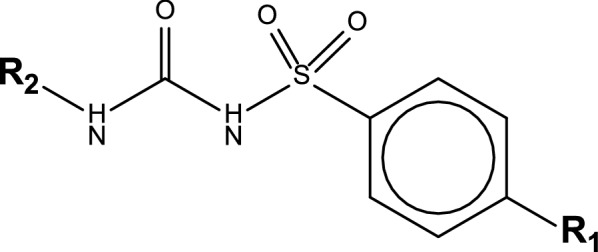
General structure of sulfonylureas, indicating S-aryl sulfonylurea, para-moiety on the phenyl ring (R1), and other substituents at N′ end terminating the urea (R2)

Sulfonylureas (SUs) comprises of the parent compound S-aryl sulfonylurea and its derivatives containing a p-substituent on the phenyl ring (R_1_) and other groups at N′ end terminating the urea (R_2_) (Fig. 1).

**Fig. 2:**
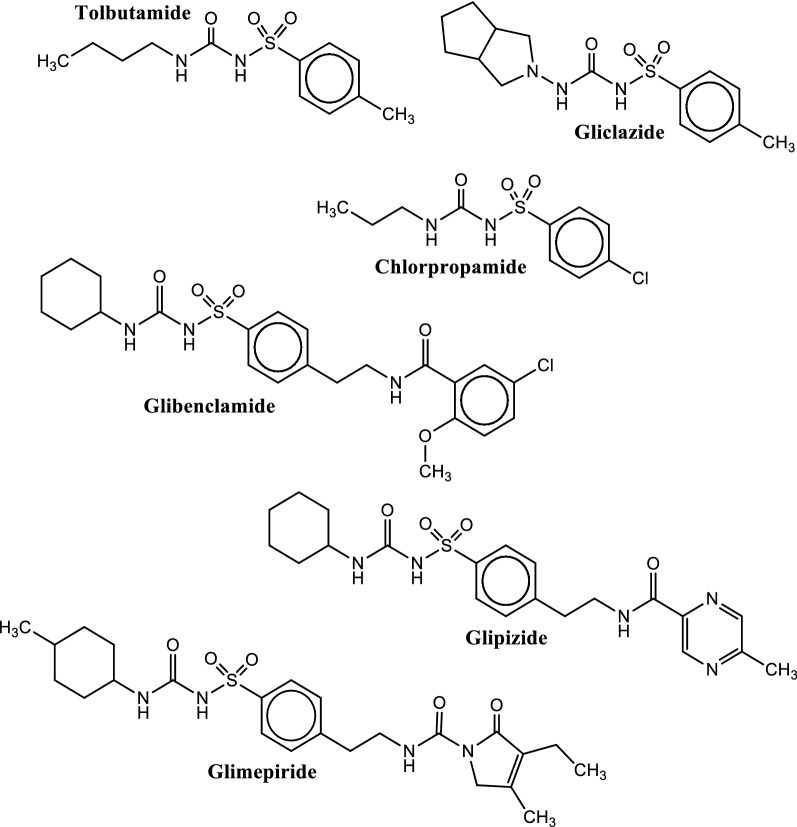
Chemical structures of some sulfonylureas

These compounds were first discovered by Marcel Janbon and co-workers in 1940s and nowadays, there are several SU drugs available in the market. These drugs are categorized into different generations based on their absorption, metabolism, toxicity, and dosing. The first generation SU drugs like tolbutamide and chlorpropamide (Fig. 2) are no longer in use, while second generation SU drugs such as glibenclamide, gliclazide, glipizide and glimepiride (Fig. 2) are currently in use as antidiabetic drugs for the management of type 2 diabetes where they increase the release of insulin from pancreatic β-cells [193]. The third generation SU drugs are currently under investigation. Glibenclamide, a second generation SU drug used in type 2 diabetes management, acts through SU receptors on pancreatic cells. These SU receptors are subunits of adenosine triphosphate-sensitive potassium channels (K^+^ATP channels), which are inhibited by glibenclamide with subsequent cell depolarization, opening of voltage-gated calcium channels, calcium influx into the cell and finally insulin secretion through vesicle exocytosis [194]. This results to the release of insulin from pancreatic β-cells into the blood stream to facilitate the uptake of glucose into peripheral cells.

#### Anticancer activity of sulfonylurea

Over the time, the antitumor effects of SUs like glibencamide and other structurally similar diarylsulfonylurea compounds have been observed since late 1980s [195]. Glibenclamide, a type of SU, has shown cytostatic effects on human breast cancer cell line MDA-MB-231 [196] and anticancer activity against non–small cell lung carcinoma tissues and cell lines [191]. However, a dose dependent risk of developing cancer was found to be associated with glibenclamide [197]. Thus, the drug should be used only in appropriate doses.

Another drug of diarylsulfonylurea group, sulofenur (LY186641), has exhibited modest antitumor activity in hematologic cancer both in in vitro and in vivo models. The antitumor effects of this drug have also been observed against ovarian cancer in phase I and II clinical trials and other several studies have reported that sulofenur, a DSU, localizes in mitochondria and causes morphological changes and cell death. Furthermore, the drug causes uncoupling of oxidative phosphorylation and reduces the level of ATP in a similar fashion as glibenclamide and other drugs of SU group [195]. Moreover, the other drugs, LY181984 and LY295501, were investigated in preclinical and clinical studies and they exhibited less toxicity in comparison to sulofenur due to their different metabolism. The improved efficiency of LY295501 as compared to other drugs of the same group tempted research for a phase I clinical study with advanced solid tumors. This drug did not exhibit any toxicity feature typically seen with sulofenur [195]. Thus, based on the available literature the less toxic SUs, like sulofenur and LY295501, can be used for treatment of ovarian cancer and advanced solid tumors, respectively. However, these drugs must undergo phase III clinical trials to determine the group of patients that will respond better to them.

Recently, 1-(anthracen-2-yl)-3-phenylurea, belonging to SU group, showed an admirable binding affinity to the SphK1 in a sub-micromolar range and significantly inhibited SphK1 activity. In addition, molecular docking study revealed that the compound fits well into the sphingosine binding pocket of SphK1 and formed significant number of hydrogen bonds and van der Waals interactions. Hence, these molecules may be exploited as potent and selective inhibitors of SphK1 that could be implicated in cancer therapeutics after the required in vivo validation [198].

Besides, various investigations have observed the increased risk of cancer with the use of SU drugs while others noted either the decreased risk of cancer or no change in risk of cancer associated with SU drugs. The existence of contradictory results on use of SUs and cancer risk may be due to the use of different SU compounds in the studies. The mechanism behind the different level of cancer risks associated with different SU compounds may be attributed to differences in the affinity of compounds with SU receptors [199].

#### Anticancer mechanism of sulfonylurea

**Fig. 3:**
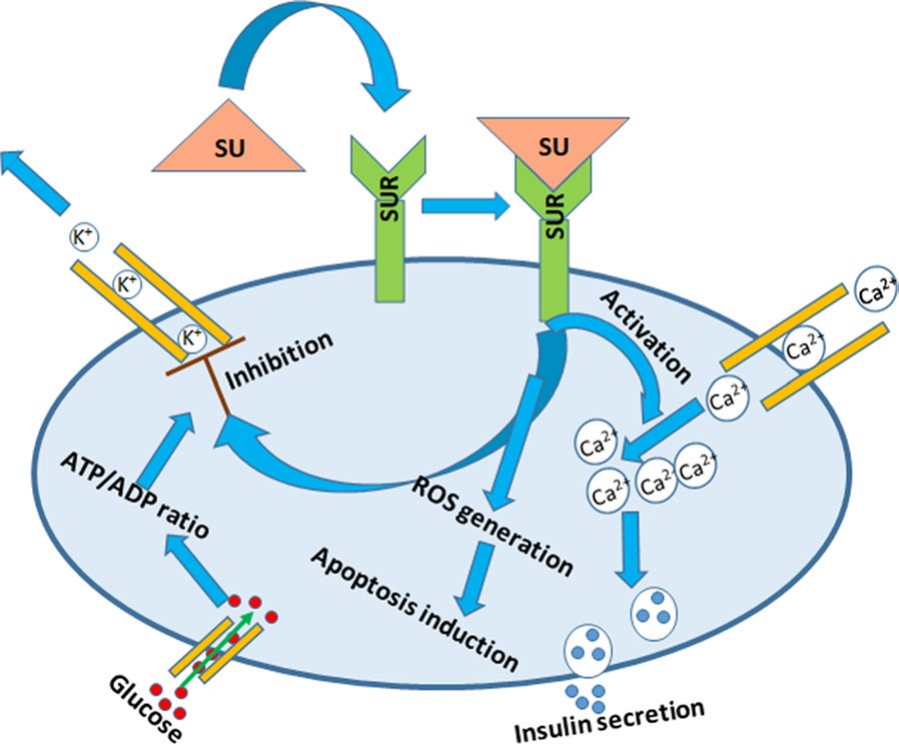
Schematic representation of anticancer and antidiabetic mechanism of sulfonylureas (SU). The binding of SU with sulphonylurea receptor (SUR) inhibits the efflux of K^+^, activates the influx of Ca^2+^ and induces the generation of reactive oxygen species (ROS). The accumulation of reactive oxygen species in turn results into apoptosis whereas increased influx of calcium (II) ions causes exocytosis of insulin by rearrangement of cytoskeleton.* ATP* adenosine triphosphate, *ADP* adenosine diphosphate

The initial work to explore the antitumor mechanism of glibenclamide was performed by a French group. They observed its role as an inhibitor of ATP binding cassette transporters [200]. These transporters belong to a group of transmembrane proteins including multidrug-resistant proteins (MRPs) and SU receptors that utilise ATP to transport many varieties of substrates across extra- and intracellular membranes including metabolic products, lipids, sterols, and drugs. This group investigated the role of glibenclamide as an inhibitor of MRP in lung cancer cells. The drug induced the accumulation of calcein, a MRP1 substrate, with overexpressed MRP1, endorsing its role as MRP1 inhibitor. Moreover, the accumulation of another MRP1 substrate, vincristine, inside the cells, indicates that glibenclamide might act as a sensitizer of cancer cells to chemotherapeutic agents. These results confirm glibenclamide as an inhibitor of ATP binding cassette transporter. However, high dose of the drug is required for MRP1 inhibition and this did not allow its clinical application. It was reported that the treatment of non–small cell lung carcinoma expressing SU receptor with glibenclamide suppresses cell growth, cell-cycle progression, epithelial–mesenchymal transition and cell migration [191]. In addition, the drug down-regulates the expression of p70S6K and up-regulates the expression of Krüppel-like factor 4, a tumor suppressor. Moreover, the ATP dependent potassium ion channels in plasma and mitochondrial cell membrane of cancer cells also comprise of SU receptors. The potassium influx through these channels promotes tumor growth and allow the cancer cells to survive in a hypoxic microenvironment through resting potential depolarization. The anticancer effect of glibenclamide on the cancer cells expressing ATP dependent potassium ion channels might be due to closure of these channels [195]. Similarly, the anticancer effect of glibenclamide was investigated in gastric cancer cell line (MGC-803) expressing ATP dependent potassium ion channel [201]. The drug was capable to induce ROS generation and apoptosis of the cells (Fig. 3). The detailed investigation of the phenomenon revealed that ROS generation activates the pro-apoptotic c-Jun N-terminal kinase and inhibits the anti-apoptotic AKT kinase enzyme activity thereby reduced the potential of mitochondrial membrane. This facilitates the release of mitochondrial cytochrome c and apoptosis-inducing factor to the cytosol which in turn could lead to caspase-dependent and independent apoptosis [201].

The synergic effects of glibenclamide and tumor necrosis factor-related apoptosis-inducing ligand in induction of cell death was examined in case of malignant pleural mesothelioma cells [195]. Increased caspase activity was observed in cells treated with both the agents as compared to untreated control and those treated with either of the agents. They also observed induced ROS in epithelioid cells treated with glibenclamide as compared to control while no changes in the level of ROS was observed in case of sarcomatoid cells. However, the reduced effects of glibenclamide were observed in case of cells pre-treated with N-acetylcysteine, a ROS scavenger. The study concluded that the malignant pleural mesothelioma cell lines and primary cultures can be sensitized to tumor necrosis factor-related apoptosis-inducing ligand-mediated apoptosis by the use of glibenclamide through different action mechanisms in different histotypes. Furthermore, it was reported [202] that glibenclamide can sensitize the melanoma cells to tumor necrosis factor-related apoptosis-inducing ligand-mediated apoptosis, possibly through depolarization of plasma membrane potential, activation of effector caspases 3 and 7, and activation of endoplasmic reticulum stress-induced caspase 12. Besides, glibenclamide also exhibited an adverse effect on invasion and migration of ovarian ES-2 cell line by angiogenesis inhibition. It was thought that the inhibition of angiogenic pathway was due to release of proangiogenic proteins and subsequent closure of ATP dependent potassium ion channel caused by the drug [203].

The above anticancer mechanisms showed that glibenclamide acts against cancerous cells through the abrogation of ATP binding cassette transporters and blockage of ATP dependent potassium ion channels and also serve as sensitizer of tumors to chemotherapeutic drugs. It results in suppression of tumor growth, cell cycle progression, cell migration, leads to generation of ROS and eventually apoptosis of cancer cells. Thus, glibeclamide could be used for patients with lung, gastric, skin and ovarian cancers.

### Biguanides

**Fig. 4:**
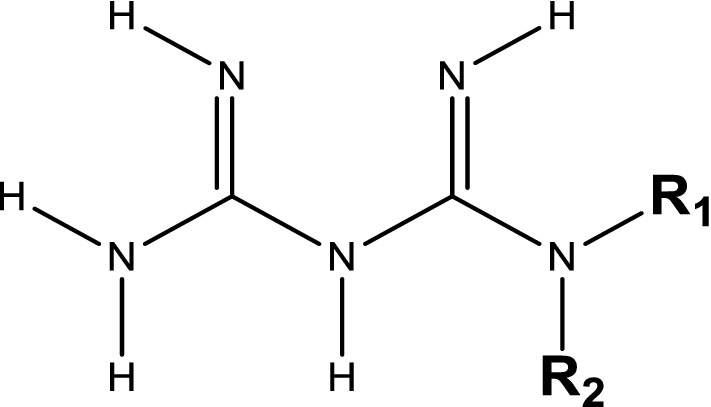
General structure of a biguanide

Biguanides are group of compounds derived from a single parent compound called guanylguanidine (biguanide) and they show hypoglycemic effect in type 2 diabetes (Fig. 4).

**Fig. 5:**
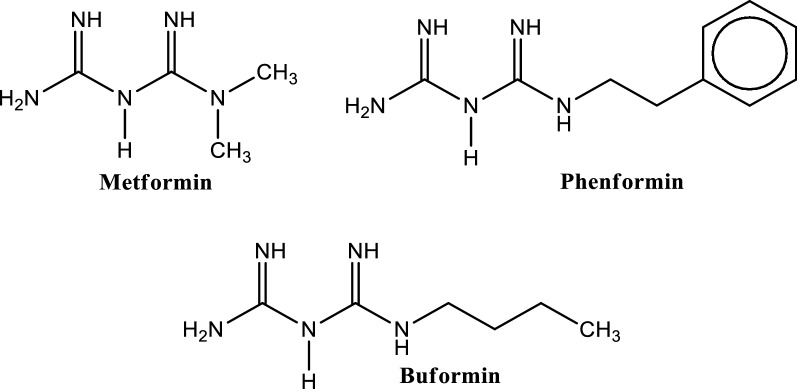
Structures of some biguanides

The commonly used biguanides are dimethylbiguanide (metformin), phenethylbiguanide (phenformin) and butylbiguaninde (buformin) (Fig. 5). Among these biguanides, phenformin and buformin were withdrawn from clinical use in various countries in the late 1970s due to the high occurrence of lactic acidosis associated with them. Metformin, which has a much lower risk of lactic acidosis, is still used widely in the treatment of type 2 diabetes [204].

Metformin is a synthetic biguanide that has been approved by United States Food and Drug Administration in 1994 and it was recommended as first line treatment for type 2 diabetes. It acts as sensitizer of insulin and can be used either alone or in combination with other drugs. Metformin may also be administered to prediabetic patients for preventing the development of diabetes and the drug is characterized by its versatile actions comprising hypoglycemic activity, impairment of hepatic gluconeogenesis, upsurge in tissue’s glucose consumption, sensitivity of insulin and reduction in intestinal glucose absorption. Additionally, it reduces the mortality rate, improves serum lipids profile, inhibits adhesion of inflammatory cell to endothelium and stimulates expression of genes responsible for antioxidant defense mechanisms in diabetic patients. Type 2 diabetes is a metabolic disorder featured by impaired blood glucose control, insulin resistance and increased insulin level in blood [205]. From clinical findings, the latter is linked with the aetiology of cancer as insulin may act as mitogen [206]. Moreover, there is substantial evidence for a direct association of type 2 diabetes and cancer, particularly in postmenopausal breast cancer [207, 208]. It is worthy to mention here that diabetic patients have 16% more risk for developing breast cancer than non-diabetic females [209].

#### Anticancer activity of metformin

Metformin is gaining global consideration for its impending use to treat or preclude various types of cancer and other diseases like cardiovascular disease, ageing & neurological disorders in addition to diabetes [210]. Mounting evidence from in vitro, in vivo and epidemiological as well as observational studies reported that metformin may be an effective treatment or helpful for the treatment of cancer. Various workers have shown that the usage of metformin does not only lowers the incidence of various types of cancer in diabetic patients [211, 212] but also decreases the mortality in patients suffering from both cancer and diabetes [213]. At the outset, a relation between the use of metformin and reduced risk of cancers and cancer-related deaths was reported [7]. Similarly, a Danish study showed lower risk of breast cancer development in peri- and postmenopausal females receiving metformin as compared to those not receiving the drug [214]. Moreover, several in vitro and preclinical studies confirmed the antineoplastic activity of metformin against several types of cancer, which prompted the onset of more than 55 clinical trials exploring the potential anticancer effect of metformin against endometrial, prostate, pancreas, lung and breast cancer [190]. A phase II clinical trial (NCT01243385) study on prostate cancer patients has shown that the administration of metformin is safe in nondiabetic patients, and it yields prostate-specific antigen responses and may induce disease stabilization. The activity of metformin in prostate cancer, along with its low cost, favourable toxicity profile and positive effect on metabolic parameters suggests that further investigation of metformin as therapy for patients with prostate cancer is of interest [215]. Besides, the completion of ongoing phase III clinical trial (NCT01905046) assessing the effect of metformin in prevention of breast cancer in patients with atypical hyperplasia or in situ breast cancer is awaiting. In overall, metformin could be effective against patients with endometrial, prostate, pancreas, lung and breast cancers. However, the drug should be tested in all the phases of clinical trials (phase I-IV) for the above-mentioned cancers to establish its effectiveness, safety and approval in the treatment of tumor.

#### Mechanism for anticancer activity of metformin

**Fig. 6:**
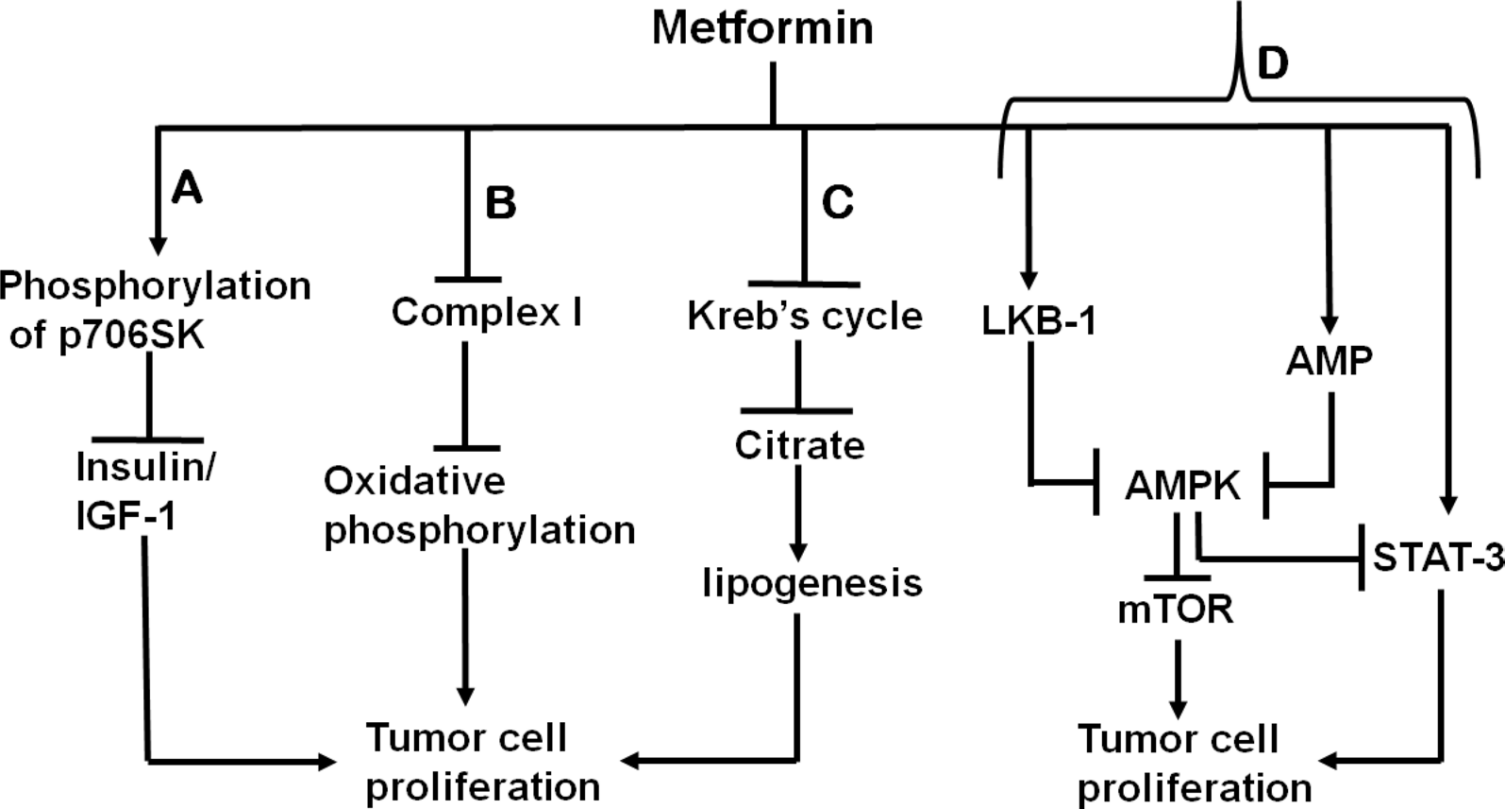
Antitumor action of metformin. The drug could inhibit the tumor cell proliferation by various mechanisms; **A** by ceasing the insulin/insulin-like growth factor 1 (IGF-1) signaling pathway, **B** by blocking the activity of complex I of the oxidative phosphorylation, **C** by interrupting the Kreb’s cycle pathway through carboxylation of α-ketoglutarate, thus inhibiting the pathway for lipogenic citrate synthesis, **D** by enhancing apoptosis via targeting AMPK and mTOR pathways or via targeting STAT-3 pathway either directly or through AMPK. *LKB-1* liver kinase B-1, *AMP* adenosine monophosphate

There is no any clear mechanism for the anticancer activity of metformin. However, various studies have suggested different mechanisms for the drug. In this sense, a group ascribed [216] the antiproliferative activity of metformin against human breast cancer to its ability to impair insulin/IGF-1-mediated signaling pathway (Fig. 6A). The authors sued that metformin could suppress the growth of insulin/IGF-1 sensitive cell by inhibiting the phosphorylation of the enzyme p70S6K. Moreover, there are ample evidences indicating that the anticancer properties of metformin are largely due to cell autonomous mechanisms which may be attributed to activity of metformin against complex I of oxidative phosphorylation (Fig. 6B) [217, 218]. However, recent data support a “substrate limitation” model according to which metformin owes its antitumor activity to the inhibition of lipogenic citrate production via the oxidative metabolic pathway in mitochondria due to drug-induced depletion of Krebs cycle intermediates in a liver kinase B1- and AMPK-independent manner (Fig. 6C).

Besides, study on activity of metformin against breast cancer cell lines showed that metformin significantly reduced both tyrosine and serine phosphorylation of STAT-3 (P-STAT-3 at Tyr705 or Ser727), reduced P-mTOR and induced P-AMPK/AMPK (Fig. 6D). Also, the study showed that metformin inhibits STAT-3 activation, either directly or indirectly, through a time- and dose-dependent manner [219] and resulting into growth inhibition. The specific knockdown of STAT-3 expression enabled metformin to significantly induce more growth inhibition of the knockdown cells. This observation concluded that the anticancer activity of metformin is achieved via direct or indirect activation of STAT-3. Further analysis of association between metformin action and AMPK revealed that metformin acts as an activator of AMPK phosphorylation, thus, AMPK and its upstream activator, the liver kinase B1 tumor suppressor, are considered to play a central role in the anticancer function of metformin [220, 221]. Previously, it was thought that activated AMPK is a negative modulator of mTOR, which is a point of conjunction for tumorigenesis [222]. However, later it was found that antitumor activities of metformin are independent of mTOR [190].

A recent study examining the effect of metformin on sphingolipid rheostat i.e. the balance of ceramide/sphingosine and sphingosine-1-phosphate in ovarian cancer [223] demonstrated that ovarian cancer patients who were using metformin for the treatment of type 2 diabetes had significantly lower serum sphingosine-1-phosphate levels than patients not using metformin. The sphingosine-1-phosphate level is believed to be regulated by sphingosine kinase which in turn regulate tumor progression [224]. A recent study on human esophageal squamous cell carcinoma suggested that cellular treatment with metformin up-regulates miR-497, which is a miRNA that was shown to be significantly down-regulated in cancer tissues [225]. Interestingly, the study suggested that proline-, glutamate- and leucin-rich protein 1 (PELP1) is a target for miR-497 and that upregulation of miR-497 will in turn down-regulate PELP1 [226]. The reduction in PELP1 causes the increase in the level of gasdermin D which in turn interacts with membrane phospholipids to form pores in the plasma membrane that eventually leads to pyroptosis. This process is a non-traditional programmed cell death characterized by pore-formation on the plasma membrane resulting in cell swelling and plasma membrane disruption. Another study investigating the anti-angiogenic effect of metformin on females with endometrial carcinoma reported that [227] preoperative metformin administration considerably reduced the expression of protein phosphatase 2A. This enzyme is considered a hallmark of antiproliferative effects of metformin administration [228]. Furthermore, the investigation on antiproliferative effect of metformin on human gastric cancer AGS cells suggested that metformin suppressed cancer cell growth via the induction of apoptosis in a concentration and time dependent manner. The study claimed that the apoptotic mechanism of metformin may involve extracellular signal regulated kinase, c-Jun N-terminal Kinase and p38 MAPK-regulated pathways in AGS cells, or through an increase in mitochondrion ROS, or through an intrinsic signaling that induces mitochondria-mediated caspase-dependent apoptosis [229]. Another recent study [230] concluded that melanoma cell growth could be suppressed after metformin treatment through impairing cell cycle progression and inducing cellular apoptosis. The next-generation sequencing (NGS) analysis of metformin treated melanoma cells have shown the upregulation/downregulation of various miRNAs and interestingly, an overexpression of miR-192-5p and miR584-3p on melanoma cell growth resulted in a clear suppression of colony formation and invasion abilities as well as proliferation, which were partly improved after miR-192-5p and miR584-3p inhibitor transfection. Furthermore, microarray analyses identified several potential target genes for miR-192-5p and miR-584-3p including the two oncogenes EFEMP1 and SCAMP3, which were significantly decreased after transfection with miR192-5p and miR-584-3p mimics, respectively. In conclusion, the results suggested that metformin treatment suppressed the motility and growth of melanoma cells due to direct modulation of miR-192-5p-EFEMP1 and miR-584-3p-SCAMP3 axes in melanoma cells. To further show the anticancer action of metformin, a group reported that overexpression of glyceraldehyde 3-phosphate dehydrogenase 1 enhances the anticancer effect of metformin through synergistic inhibition of mitochondrial function [231]. In this study, workers have observed that the treatment of metformin in the cells overexpressing glyceraldehyde 3-phosphate dehydrogenase 1 enhanced the level of ROS and damage the mitochondrial structure, which may ultimately lead to cell death. The effect of nutritional environment on the anticancer activity of metformin using renal cancer cells [232] was investigated, where treatment with metformin under normal conditions resulted in a significant suppression of cell growth, but change in the cellular environment, from normal to glucose-deprived, reversed the metformin-induced growth suppression. Thus, metformin appears to promote cell growth under this condition. However, in another study to investigate the anticancer activity of metformin on human ovarian cancer cells [233] revealed that metformin treatment in low glucose environment enhances ovarian cancer cell cytotoxicity by apoptosis induction via mitochondrial pathway, evident by the increased ratio of B-cell lymphoma-2-associated X protein/B-cell lymphoma-2 (Bax/Bcl-2). Hence, these studies also evidenced the anticancer action of metformin that resulted in growth suppression and apoptosis of cancerous cells especially of renal and ovarian origin.

### Thiazolidinediones

**Fig. 7:**
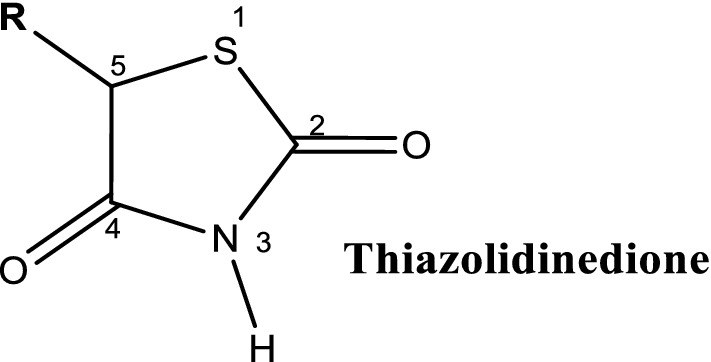
Structure of thiazolidinedione

Thiazolidinediones (TZDs), also called glitazones, are five-membered carbon ring molecules containing two heteroatoms, nitrogen and sulphur. One carbonyl group in the thiazole at position 4 and another at position 2 make the heterocyclic compound a thiazolidine-2,4-dione (Fig. 7). These compounds are synthetic ligands of peroxisome-proliferator-activated receptor gamma (PPARγ) nuclear receptors.

**Fig. 8:**
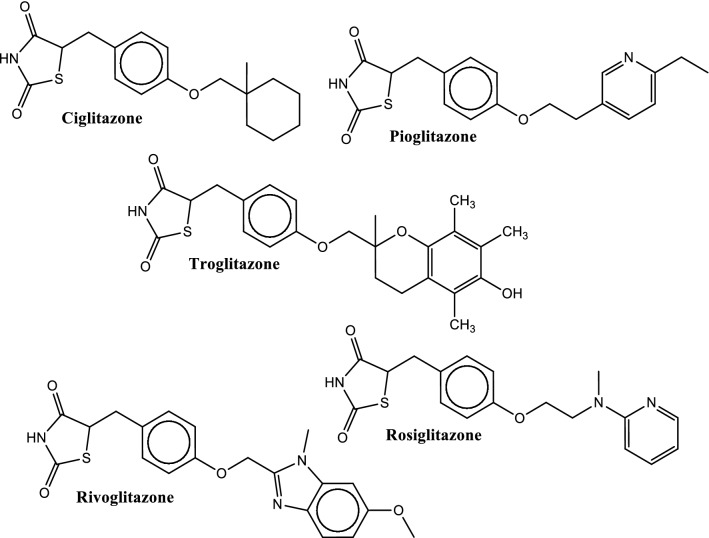
Structure of some thiazolidinediones

The prototype of TZDs is ciglitazone (Fig. 8) which could never be approved for clinical use due to its poor bioactivity. Till date, only three TZDs; troglitazone, rosiglitazone, and pioglitazone (Fig. 8) have been clinically approved for the treatment of type 2 diabetes [234]. The first TZD, troglitazone, showed beneficial effects on glucose levels, insulin sensitivity and free fatty acid concentration, but the drug was withdrawn from market in 2000 because of its severe hepatotoxicity. The second antidiabetic TZD, rosiglitazone, which was clinically approved has now been under controlled use in USA and banned in Europe due to its cardiovascular morbidity. The use of third TZD, pioglitazone has also been suspended in 2011 by French and German medicine agencies due to concerns regarding risks of bladder cancer development by the use of this drug and the fourth antidiabetic TZD, rivoglitazone (Fig. 8), is still under investigation [235]. The restrictions and withdrawals of TZDs from the markets seems to be due to the highly pleiotropic action of these PPARγ inhibitors and crosstalk of PPARγ with other signaling pathways. All the TZDs are capable of activating PPARγ, a receptor and a member of the nuclear receptor superfamily of transcription factors [236, 237]. The activation of PPARγ receptor promotes secretion of adiponectin and uptake of fatty acids by adipocytes. It also supresses inflammatory responses involved in insulin resistance and thereby improves insulin sensitivity. However, a number of studies have evidenced that the treatment with TZDs for long term can lead to increased risk of obesity, cardiac diseases and cancer [238–240]. The adverse effects of TZDs are not common for all the drugs belonging to the TZD group, but they are compound specific. This can be easily comprehended by the fact that troglitazone leads to massive hepatic necrosis while pioglitazone leads to increased risk of bladder cancer [197].

#### Anticancer activity of TZDs

The idea of using TZDs for treatment of cancer has originated from the fact that the expression level of PPARγ nuclear receptors differs in the normal and transformed tissues, and are involved in cell proliferation [241]. The anticancer activity of TZDs have been reported in various types of cancer [242]. In addition, some TZDs are reported to increase the sensitivity of cancer cells for standard anticancer drugs [243]. All the three TZDs, troglitazone, rosiglitazone, and pioglitazone, approved for treatment of type 2 diabetes are reported to exhibit antitumor effects in preclinical and clinical studies [234]. The other TZD derivatives like efatutazone and netoglitazone have also been reported to show antitumor effects [241]. However, no antiproliferative effects of TZDs were observed in several similar studies [197]. Thus, no uniform results have been observed for antiproliferative effects of TZDs in various in vitro and clinical studies [232, 244]. The promising potential of three TZD derivatives, AC18, AC20, AC22, as antiproliferative agents for the treatment of prostate and breast cancer was also highlighted [192]. These compounds significantly reduced viability and migration of MCF-7, and PC3 cells in vitro and their effects were even more pronounced when compared with rosiglitazone, a well-known member of the TZD class of antidiabetic agents. Despite the requirement of more research to confirm the efficacy and safety, the preclinical and clinical studies identified these compounds as potential leads for development of novel adjuvant tool for treatment of prostate and breast cancer. Recently, the in vitro antiproliferative activity of two TZDs, 5-(4-methoxybenzylidene)-3-((5-(2-chlorophenyl)-1,3,4-oxadiazol-2-yl)methyl)thiazolid, ine-2,4-dione, 5-(4-methoxy-benzylidene)-3-((5-(4-bromophenyl)-1,3,4-oxadiazol-2-yl)methyl)thiazolid ine-2,4-dione, by inhibition of enzyme thymidylate synthase, a vital enzyme in DNA synthesis and proliferation of cancer cells, was also reported [245].

#### Mechanism of antitumor action by TZDs

**Fig. 9:**
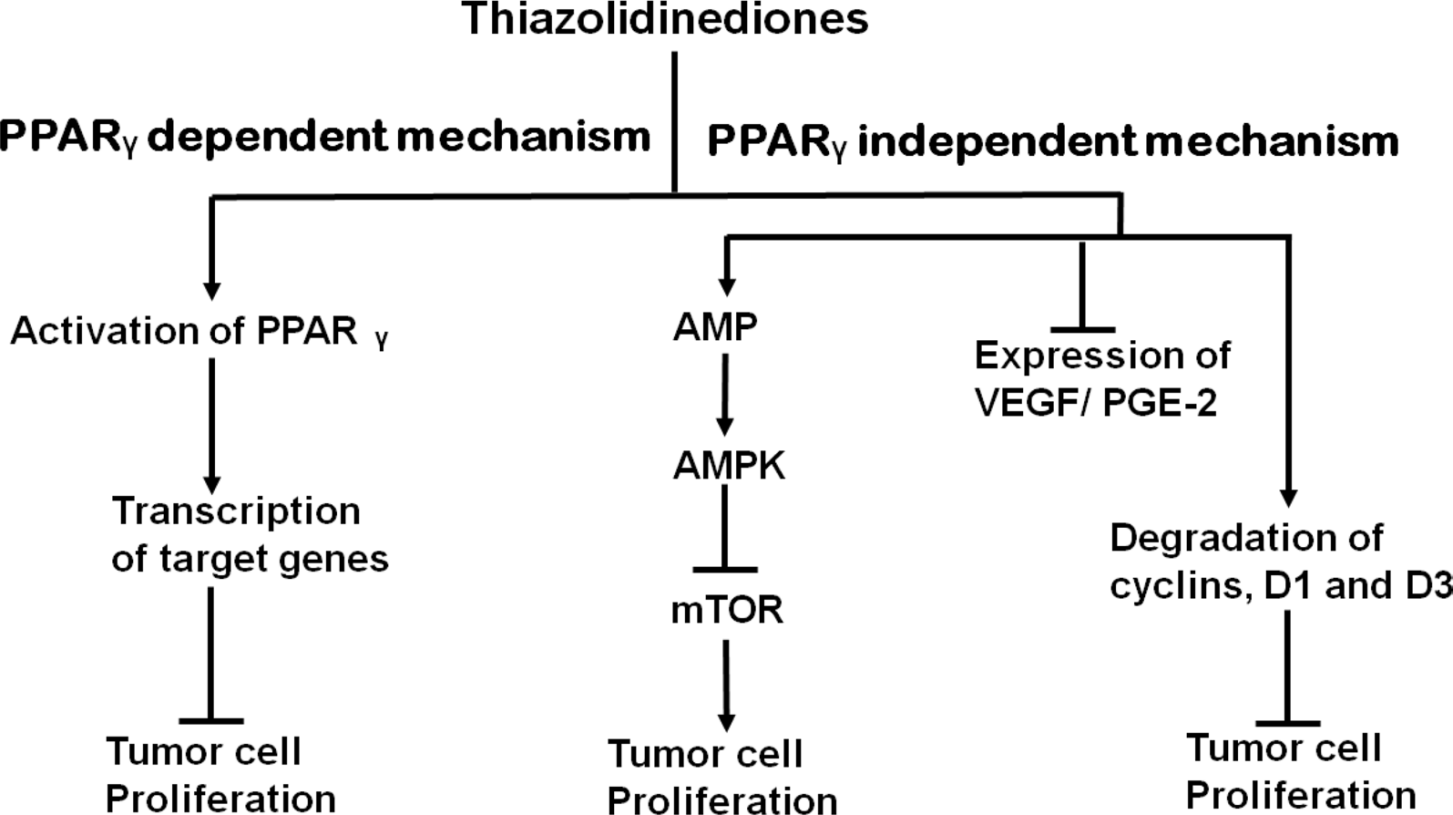
Antitumor mechanism of Thiazolidinediones (TZDs). These drugs inhibit the proliferation of tumor cells either through peroxisome-proliferator-activated receptor gamma (PPARγ)-dependent or PPARγ-independent mechanisms. In PPARγ-dependent mechanism, the activation of PPARγ receptor by these drugs switch on the transcription of various target genes which in turn help in suppressing the growth of tumor. In PPARγ- independent mechanisms TZDs blocks the mTOR pathway by activation of AMPK), inhibits the expression of prostaglandin E2 (PGE2) receptor and vascular endothelial growth factor (VEGF) genes as well as induction of cyclins degradation. *AMP* adenosine monophosphate

Different compounds of thiazolidinedione group exhibit different mechanism for their anti-tumor action, for instance, ciglitazone stimulates the expression of p21 and suppress the cyclin D1 by PPARγ independent pathways, while rosiglitazone acts through PPARγ dependent pathway to persuade the same effects in androgen-independent prostate carcinoma cells [246] (Fig. 9). The PPARγ dependent antitumor effects of TZDs can be explained by genomic activation or transactivation of PPARγ nuclear receptors. Briefly, the TZDs act as ligand and activate the PPARγ receptor by inducing a conformational change, thereafter, the activated receptors form heterodimers with the retinoid X-receptor. This peroxisome-proliferator-activated receptor/retinoid X-receptor complex binds with PPARγ response element in target genes and activates the transcription of these genes [247], which in turn, leads to reduced proliferation, relocation and inflammation whereas causes increased differentiation and apoptosis. Anti-inflammatory effect of PPARγ were observed due to inhibition of TNF-α, IL-1β, IL-6 and prostaglandin E_2_ production [248]. Also, transactivation of PPARγ lowers the level of angiogenic factors and reduces the migration and proliferation of endothelial cells [249]. The overexpression of PPARγ receptors in SNU-668 gastric cancer cells having adenovirus gene exhibited significant growth inhibition and activation of apoptosis due to strong up-regulation of a tumor suppressor gene, insulin-like growth factor-binding protein-3 [250]. Some workers have shown that the genomic activation of PPARγ by microRNA-125b suppress the expression of B-cell lymphoma 3 protein, a proto-oncogene, thereby reduce the growth of ovarian cancer [251]. Besides, the phenomenon of PPARγ transactivation promotes tumor necrosis factor-related apoptosis-inducing ligand-induced apoptosis in human lung cancer via autophagy [252]. It also contributes to the pro-apoptotic phenotype of cancer cells; however, the molecular mechanism of this process is still unknown [247].

PPARγ-independent mechanisms for antitumor activity of TZDs depend on the increased expression of PTEN/AMPK, reduced expression of Akt/mTOR as well as proteosomal degradation of cyclins, D1 and D3. Further, these drugs act by inhibiting the expression of target genes such as prostaglandin E_2_ receptor gene, insulin receptor gene and vascular endothelial growth factor gene [192]. Besides, ciglitazone is known to down-regulate the aromatase activity in androgen dependent prostate carcinoma [253].

## Use of antidiabetic drugs and cancer risk

Antidiabetic agents such as biguanides, SUs and TZDs are most commonly used drugs around the world consumed by about 0.35 billion diabetic people. Their use as antitumor agents have been discussed in previous sections but their safety is still a major concern for scientists as there are several other studies which have shown that few antidiabetic drugs might increase the risk for certain cancers. Therefore, the beneficial roles of antidiabetic agents in cancer management and their risk in cancer development and progression still remain a subject of controversy. The effect of metformin, sulfonylureas and insulin on the risk of various cancers have been analysed and derivatives of sulfonylurea and insulin exposure have been documented to increase the risk of cancer [262]. But, they indicated that metformin usage as combined or single therapy did not show any risk of malignancy development. Contrary to this, some authors did not document protective role of metformin on the development of cancer [255]. Also, monotherapy of SU or insulin showed elevated risk of malignancy development, however, combined therapy with metformin abolishes the adverse effect on cancer. The authors proposed that poor metabolic regulation in insulin monotherapy was responsible for the prevalence of cancer and this is associated with the duration of diabetes. The mechanisms here include insulin’s direct and indirect effect on the growth of cancer. The hormone, which is an active growth stimulating hormone, acts on type A insulin receptor resulting to its stimulation and enhancement of cancer proliferation [256, 257]. Moreover, hyperinsulinemia (resulting from subcutaneous insulin injection) stimulates hepatic expression of IGF-1 and elevates its bioavailability via depletion of IGFBP-1/2 [254]. Consistently, SU was proposed to stimulate carcinogenesis by elevating the activity of IGF-1, which results into impaired activation of different cellular signaling pathways, promoting growth factor-linked cell proliferation and affecting cellular metabolism [258].

A population-based investigation analysing the effects of antidiabetic medications on the risk of pancreatic cancer in Korean patients has concluded that the exposure of sulfonylurea and insulin was related to increased risk of pancreatic cancer compared to subjects with no drug exposure [259]. In another study, the administration of pioglitazone, insulin and its analogues to the diabetic patients were found to be associated with increased risk of pancreatic cancer by 45%, hepatic cancer by 32% and pulmonary cancer by 18% as compared to the non-users. However, in the same study, metformin, glibenclamide, acarbose and others did not show any evidence of association with cancer risk [232]. Thus, it is clear that each compound of insulin, sulfonylureas and thiazolidinedione  group could be assessed for risk of various types of cancer before its use as antidiabetic or anticancer medication. However, the metformin may be recommended as a safe antidiabetic medication with anticancer properties. Based on the aforementioned data, some antidiabetic drugs such as pioglitazone could serve as risk factor for pancreatic cancer, among the types of cancers.

## Possible mode of actions of Antidiabetic drugs for cancer risk

A plethora of studies have tried to decipher this area of investigation and attention has been drawn to their limitations. In a case–control study for investigating the association between antidiabetic medication and cancer risk over 20 years, it was reported that, only pioglitazone and insulin analogues as antidiabetic drugs were associated with cancer risk [260].

The use of pioglitazone, and not rosiglitazone, has been associated with an increased risk of bladder cancer in a population-based cohort study, suggesting the risk is TZD specific and not a particular class. It has been reported that pioglitazone associated prolonged and higher PPARγ activity levels are associated with higher incidences of bladder cancer. Several mechanisms accounting for the same are: the downstream effects of PPARγ-mediated metabolism (by altering the microenvironment that allows the cells to autonomously synthesize nutrients through lipid accumulation and angiogenesis) and increased cancer cell migration and invasion [197]. Moreover, peroxisome proliferators also act as a driving force to malignancy by inducing oxidative stress, VEGF expression, COX-2 expression, & PGE_2_ production and inhibiting apoptosis [249].

Insulin and its analogues may function as growth factors and therefore have a theoretical potential to promote tumor proliferation through various mechanisms involving activation of the insulin receptor, IGF-1 receptor (IGF-1R) and extracellular-signaling-regulated kinase (ERK) pathways [261]. Studies on in vitro models indicate that in contrast to long-acting analogues, short-acting analogues elicit molecular and biological effects similar to those of insulin.

Regarding usage of insulin, various reports and data related to cancer risk are there but still inconclusive as plethora of factors are needed to study for a meaningful comparison. For instance, one of the major issues is that clinical decisions deciding each patient’s treatment are not random and people are prescribed with different therapies for number of health-associated reasons. Therefore, health outcomes might vary between people taking different therapies even if the therapies themselves have no such effect [261].

Interestingly, during the subclinical phase, insulin requirements might be affected by the undetected cancer leading to changes in treatment, thus appear to be favouring cancer for an unwary observer. Whereas, vice versa it is cancer that produces treatment change [262]. As already mentioned, the available clinical evidence thus can neither demonstrate nor exclude an increased risk of cancer in diabetic patients treated with insulin analogues.

Therefore, prospective clinical studies are needed to evaluate the possible tumor growth-promoting effects of these insulin analogues.

## Conclusion

Diabetes and cancer are two disorders with related metabolic links. Antidiabetic drugs such as sulfonylureas, biguanides, and thiazolidindiones exhibited repurposing actions in cancer management and this is attributed to the metabolic links (this includes hyperglycemia, hyperinsulinemia, inflammation, and oxidative stress, among others) between the two diseases. The repurposing actions of antidiabetic drugs in the management of cancer serve as an alternative intervention for alleviating some of setbacks produced by anticancer agents. Although, the use of antidiabetic drugs can serve as a risk factor for the development of cancer, however, their beneficial roles in cancer management overcomes their unwanted effects.

## Data Availability

Yes.

## Abbreviations

AGE: Advanced glycated end product
AMPK: Adenosine monophosphate-activated protein kinase
ATP: Adenosine triphosphate
BCL: B-cell lymphoma
DNA: Deoxyribonucleic acid
EGFR: Epidermal growth factor receptor
HCC: Hepatocellular carcinoma
IGF: Insulin-like growth factor
IGFBP: IGF binding protein
IL: Interleukin
IR: Insulin receptor
IRS-1/2: Insulin receptor substrate ½
JAK: Janus kinase
LRP5/6: Low-density lipoprotein receptor-related protein 5 and 6
MAPK: Mitogen-activated protein kinase
MRP: Multidrug resistant protein
mTOR: Mammalian target of rapamycin
mTORC1/2: Rapamycin sensitive mTOR complex ½
NF-κB: Nuclear factor-kappa B
PI3K: Phosphoinositide 3-kinase
PTEN: Phosphatase and tensin homolog
RAGE: Receptor for advanced glycation end product
RNA: Ribonucleic acid
RNF43: Ring finger 43
ROS: Reactive oxygen species
PPARγ: Peroxisome-proliferator-activated receptor gamma
STAT: Signal transducer and activator of transcription
SU: Sulfonylurea
TNF-α: Tumor necrosis factor alpha
TSC: Tuberous sclerosis complex
TXNIP: Thioredoxin-interacting protein
TZD: Thiazolidinedione
ZNRF3: Zinc and ring finger 3/43
